# Water-induced thermogenesis and fat oxidation: a reassessment

**DOI:** 10.1038/nutd.2015.41

**Published:** 2015-12-21

**Authors:** N Charrière, J L Miles-Chan, J-P Montani, A G Dulloo

**Affiliations:** 1 Department of Medicine, Division of Physiology, University of Fribourg, Fribourg, Switzerland

## Abstract

**Background/Objectives:**

Drinking large amounts of water is often recommended for weight control. Whether water intake stimulates energy and fat metabolism is, however, controversial with some studies reporting that drinking half a litre or more of water increases resting energy expenditure (REE) by 10–30% and decreases respiratory quotient (RQ), whereas others report no significant changes in REE or RQ. The aim here was to reassess the concept of water-induced thermogenesis and fat oxidation in humans, with particular focus on interindividual variability in REE and RQ responses, comparison with a time-control Sham drink, and on the potential impact of gender, body composition and abdominal adiposity.

**Subjects/Methods:**

REE and RQ were measured in healthy young adults (*n*=27; body mass index range: 18.5–33.9 kg m^−2^), by ventilated hood indirect calorimetry for at least 30 min before and 130 min after ingesting 500 ml of purified (distilled) water at 21–22 °C or after Sham drinking, in a randomized cross-over design. Body composition and abdominal fat were assessed by bioimpedance techniques.

**Results:**

Drinking 500 ml of distilled water led to marginal increases in REE (<3% above baseline), independently of gender, but which were not significantly different from Sham drinking. RQ was found to fall after the water drink, independently of gender, but it also diminished to a similar extent in response to sham drinking. Interindividual variability in REE and RQ responses was not associated with body fatness, central adiposity or fat-free mass.

**Conclusions:**

This study conducted in young men and women varying widely in adiposity, comparing the ingestion of distilled water to Sham drinking, suggests that ingestion of purified water *per se* does not result in the stimulation of thermogenesis or fat oxidation.

## Introduction

Since the attribution, in the early 1980s, of a pivotal role of the sympathetic nervous system (SNS) in the control of thermogenesis pertaining to weight regulation, bioactive food and beverage ingredients with sympathomimetic effects have been the focus of considerable interest for their potential thermogenic, fat-oxidising and anti-obesity properties.^1, 2, 3^ These include caffeine, catechin polyphenols, capsaicinoids and medium-chain fatty acids—all of which have been shown to stimulate thermogenesis and/or fat oxidation in humans either by increasing SNS activity and/or potentiating its actions on cellular metabolism. In this context, the findings that drinking a large glass of water also increases sympathetic activity, as measured by increased plasma noradrenaline concentrations,^4^ and enhances sympathetic neural activity to skeletal muscle^5^ have led to the concept that water drinking might be strategy to stimulate thermogenesis for weight control.

The proof of concept for water-induced thermogenesis was first claimed by Boschmann *et al.*^6^ who reported that the ingestion of half a litre of water led to a relatively large increase in resting energy expenditure (REE) of about 30% between 30 and 90 min post drink in both men and women. From these results, Boschmann *et al.*^6^ proposed that increasing water intake by 1.5 litre per day could lead to an increase in energy expenditure of 200 kJ per day, thereby underscoring quantitatively important thermogenic potential of water drinking for weight control. These findings of water-induced thermogenesis in normal weight adults^6^ were subsequently extended to overweight and obese adults in whom 500 ml of water was reported to increase REE by 24% over 60 min post drink.^7^ In more recent years, two other laboratories have also reported substantial increases in REE after water drinking. Kocelak *et al.*^8^ found that the ingestion of a litre of water increased REE by 12 and 20% (over 1 h post drink) in normal weight and obese women, respectively. Dubnov-Raz *et al.*^9^ reported an increase in REE by up to 25% in overweight children after drinking cold water given as 10 ml kg^−1^ body weight, that is, after an absolute water intake in the range of 340–800 ml.

The concept of water-induced thermogenesis is, however, controversial. Indeed, several past studies of human energy metabolism where water has been used as a control drink,^10, 11, 12, 13, 14, 15, 16, 17, 18^ and a few more recent ones,^19, 20^ suggest that water drinking has little or no effect on REE (Table 1). Several reasons can be put forward to explain these discrepancies,^19^ including differences in protocols across laboratories, different indirect calorimetry methodologies, different water loads and the type of water ingested. Furthermore, discrepancies in the literature can also be found for the effect of water drinking on the respiratory quotient (RQ). Although some studies have reported no effect of water drinks on RQ,^9, 11, 13, 16, 19^ others have shown a decrease in RQ^6, 8, 14, 18^ that has been interpreted as a fat-oxidizing property of water drinking.^6, 8^ None of the latter studies, however, refer to an appropriate time-control experiment as it is possible that with continued fasting, one may expect an increase in fat oxidation (that is, decreased RQ) independently of water intake. Finally, another potential source of discrepancy across studies could reside in the low number of subjects, particularly in studies failing to detect a significant effect of water drinking on REE. Given the large interindividual variability that have been reported for thermogenic responses to foods and beverages in general,^21, 22^ and that ‘good burners' seem to be much less prevalent than ‘poor burners',^23, 24^ small sample size increases risks for ‘random' selection biased towards those with a low capacity for thermogenesis.

In a reassessment of the concept of water-induced thermogenesis and fat oxidation reported here, we have conducted experiments involving the ingestion of 500 ml of purified distilled water (DW) in a relatively large population sample of young adults, with particular focus on interindividual variability in REE and RQ responses, comparison with a time-control Sham drink, and on the potential impact of gender, body composition and abdominal adiposity.

## Materials and methods

### Subjects and study design

The study was conducted in 27 healthy young adults (14 men and 13 women), with a mean (±s.e.m.) age of 25±1 years, weight of 74.1±2.9 kg and body mass index (kg m^−2^) of 24.8±0.7 (range 18.5–33.9). Each subject completed two separate test days involving the ingestion of 500 ml of room-tempered (21–22 °C) DW or Sham drinking, according to a randomized cross-over design, with at least 2 days interval between test days; the Sham drinking is performed with the subject raising a glass containing 500 ml of DW to their lips but without ingesting the water. We used a fixed volume of water (500 ml) and water temperature (21–22 °C) in the context of our reassessment of the findings of Boschmann *et al.*^6, 7^ for large effects of water drinking on thermogenesis when ingesting the same volume (500 ml) of water at similar (22 °C) water temperature. Measurements in women were made during the follicular phase of their menstrual cycle. Smokers, claustrophobic individuals, individuals taking medication and those with any metabolic disease were excluded. The study complied with the Declaration of Helsinki and was approved by the institute's ethical review board; all participants gave written consent. The study is registered under trial registration number of ISRCTN 57611296.

Between 2 and 5 days before first test day, the participants visited the laboratory to complete a questionnaire regarding their lifestyle and medical history, and to familiarize themselves with the experimental procedures and equipment. After voiding the bladder, body weight and height were measured using a mechanical column scale with integrated stadiometer (Seca model 709, Hamburg, Germany), body composition using a multi-frequency bioelectrical impedance analysis (Inbody 720, Biospace Co., Ltd, Seoul, Korea), and waist circumference and abdominal fat percentage by bioelectrical impedance analysis using ViScan (Tanita Corporation, Tokya, Japan), which has been shown to be accurate both for the measurement of waist circumference^25^ and for predicting total abdominal fat when validated against Magnetic Resonance Imaging techniques.^26, 27^

All participants were requested to avoid physical activity, caffeine and dietary supplements in the 24 h before testing. Furthermore, to minimize the effect of physical activity on the morning of each test day, participants were requested to use motorized transport instead of walking or cycling to reach the laboratory.

### Test protocol

On the test days, participants arrived at the laboratory at about 8 h following a 12-h overnight fast. After voiding the bladder, they rested for about 15–20 min in a comfortable car seat adapted for ventilated hood indirect calorimetry as described previously.^28^ Continuous measurements of REE and RQ were then made using the Cosmed system (Quark RMR, Cosmed, Rome, Italy) for 30–35 min, during which stabilization of REE was achieved during the last 15 min at least. Stabilization was defined as no >2% variability of REE, with no consistent upward or downward trend, as previously described.^29^ The hood was removed and the subjects ingested 500 ml of DW (or performed sham drinking) over a period of 3 min. The hood was then put back again, and gas exchange measurements were continued for a further 130 min.

### Data and statistical analysis

Power analysis, using the web software (http://www.statisticalsolutions.net/pss_calc.php) with type-I error (*α*) of 0.05 and a desired power (1−*β*) of 0.90, suggests that 11 subjects would be required to show a 4% increase in REE after water ingestion and a s.d. (*σ*) of 0.2 kJ min^−1^ (19). Randomization was performed by computer-executed software (https://www.randomizer.org/). Data are provided as mean±s.e.m. Statistical analysis was performed by analysis of variance for repeated measures with time and drink as within-subject factors using statistical software (Statistix version 8.0; Analytical Software, St Paul, MN, USA), and *post hoc* Dunnett's multiple comparison test was used to determine post-drink changes over time from baseline. For all tests, significance was set at *P*<0.05.

## Results

**Figure 1:**
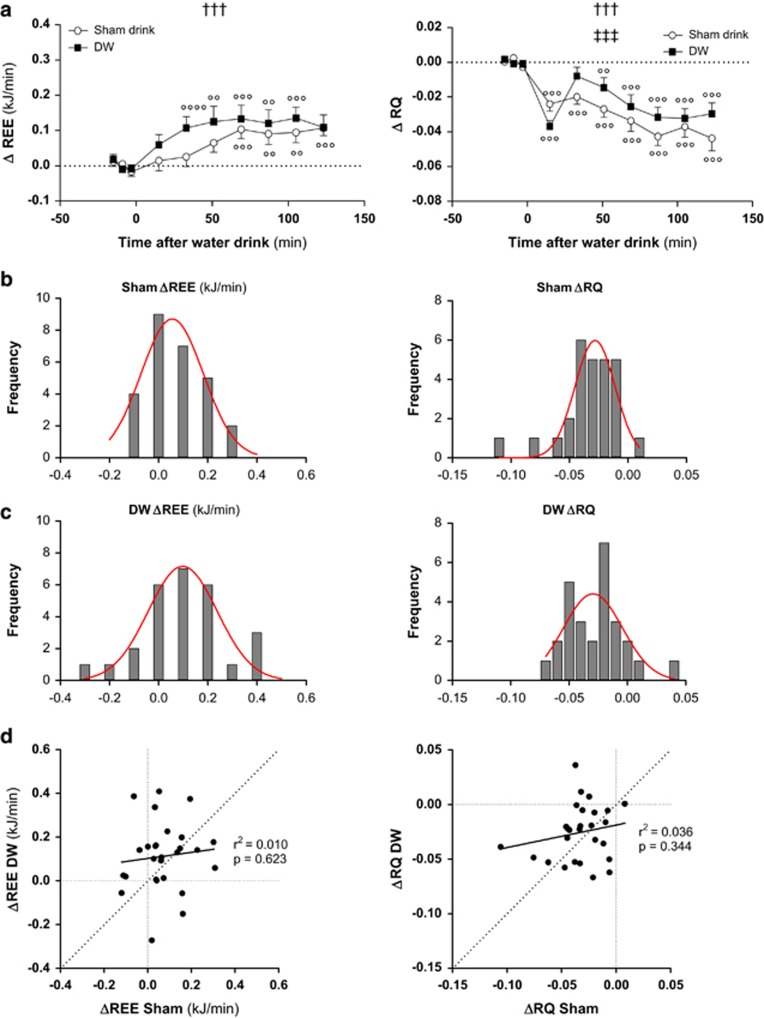
(**a**) Time course of changes in REE and RQ after Sham drink or DW in 27 young adults. Repeated-measures analysis of variance assessed statistical differences as follows: effect of time (symbol †) and the drink x time interaction (symbol ‡); one, two and three symbols denoting *P*<0.05, *P*<0.01 and *P*<0.001, respectively. Significant difference between post-drink and baseline values are indicated as follows: °*P*<0.05; °°*P*<0.01; °°°*P*<0.001). (**b**–**d**) Frequency distribution of changes in REE and RQ after Sham drinking (**b**) and DW drinking (**c**); the red colour line being the normal curve. In **d**, the individual values are plotted for changes after DW drinking versus after Sham drinking; the dotted diagonal line being the line of identity, and the solid line being the regression line (*r* values are not significant).

The mean pre-drink baseline values for REE and RQ for all subjects, as well as within each gender are shown in the Table 2; and indicate no significant difference between test days in any of these pre-drink values. The temporal changes in REE and RQ over 130 min after the DW drink or Sham drink are presented in Figure 1a (left-hand side). Overall, there is a small but significant increase in REE values after both DW and Sham drink (*P*<0.001). Although the increase in REE after DW tends to be greater than for Sham drink, this difference is not statistically significant at any time point nor when averaged over the entire 130 min post-drink period (+1.5% for Sham versus 2.7% for DW). There is a rather large (overlapping) interindividual variability in the change in REE after DW drink (range of −0.3 to 0.4 kJ min^−1^) and after Sham drinking (range of −0.1 to 0.3 kJ min^−1^). As shown in Figures 1b and c (left-hand side), the frequency distribution of changes in REE after DW is not different compared with that for Sham drinking, and furthermore, there is no correlation between the changes in REE after DW versus Sham drinking (Figure 1d).

The time course of RQ after the DW drink, shown in Figure 1a (right-hand side), indicates that RQ showed an initial transient drop within the first 45 min post drink, followed by a gradual drop till 90 min post drink. However, Sham drinking also led to a gradual and significant fall in RQ, albeit without the evident initial transient change. No significant differences are observed between DW and Sham drink for changes in RQ when averaged over the entire post-drink period (−0.028 versus −0.31, respectively); the interindividual variability in the change in RQ was in the range of −0.07 to 0.04 after DW drink, and in the range of −0.11 to 0.01 kJ min^−1^ after Sham drinking. As shown in Figures 1b and c (right-hand side), the frequency distribution of changes in RQ after DW is not different compared with that for Sham drinking, and furthermore there is no correlation between the changes in RQ after DW versus Sham drinking (Figure 1d).

Analysis of the data for potential gender differences indicates no significant differences between men and women in their REE or RQ responses to DW or to Sham drink (Supplementary Information, section A). Although within each gender, there is a tendency for the increase in REE to be higher with DW than with Sham drink, these differences are small and not statistically significant, and within each drink type, men and women did not differ significantly in their changes in REE or RQ. Furthermore, no significant correlation can be observed between the change in REE or in RQ and various parameters of body composition (total body fat%, abdominal fat%, fat mass and fat-free mass) whether for data in response to DW or to Sham drinking.

## Discussion

The main findings of this study investigating the acute metabolic effects of water *per se*, that is, in purified form as DW, can be summarized as follows:

- Drinking 500 ml of DW led to marginal (<3%) increases in REE, independently of gender, and the REE response to the water load was not significantly different when compared with that observed after Sham drinking.
- RQ was consistently shown to fall after the DW drink, independently of gender, but it also diminished to a similar extent in response to Sham drinking.
- The interindividual variability in REE and RQ response was not associated with body fatness, central adiposity or parameters of lean body mass.

### Water-induced thermogenesis: a Sham effect

The present study on the basis of a total of 27 subjects, therefore, reinforces the conclusion reached in a previous study from our laboratory, albeit conducted on a much smaller sample size (*n*=8), that the ingestion of DW has little or no significant effect on REE.^19^ It also provides evidence that extends this conclusion to both men and women, as well as across a wide range of body mass index and body composition. Although REE relative to baseline was significantly increased by 2.7% on average, Sham drinking also produced a small increase in REE of 1.5% on average over the same post-drink time period. Indeed, no significant difference can be demonstrated in REE after DW compared with REE after Sham drinking, both in men and women, suggesting that the ingestion of the water load *per se* does not lead to the stimulation of thermogenesis. A possible explanation for the small increase in REE after DW, also found in response to the Sham drink, could reside in the psychobiological, and perhaps sensorial, aspects related to the act associated with drinking (that is, without actually ingesting the water) rather than the effect of the ingested water load *per se*. In another experiment conducted on 17 young men, the increase in REE after ingestion 500 ml of DW was even smaller (+1% on average, not significant), and in 10 men who repeated the DW drinking test on 2 separate days, the lack of significant difference in their REE response to DW was confirmed on both days (Supplementary Information, section B).

### Water-induced fat oxidation: a sham effect

The importance of Sham drinking in the interpretation of the reduction in RQ after the water drink is underscored in our study here. We found significant reductions in RQ with time after the water drink, but also after Sham drinking. We did not directly measure fat metabolism, but fasting RQ has often been observed to fall over time in the absence of any intervention,^30^ and it has been demonstrated that the prolongation of the conventional 12-h overnight fast by 6 h result in elevated plasma FFA concentrations and increases in net lipid oxidation.^31^ Thus, the drop in RQ in response to water ingestion, like for sham drinking, is most likely explained by the continued fasted state of the subject rather than to an effect of the ingested water load *per se* on fat oxidation.

### Interindividual and inter-laboratory variability in REE

**Figure 2:**
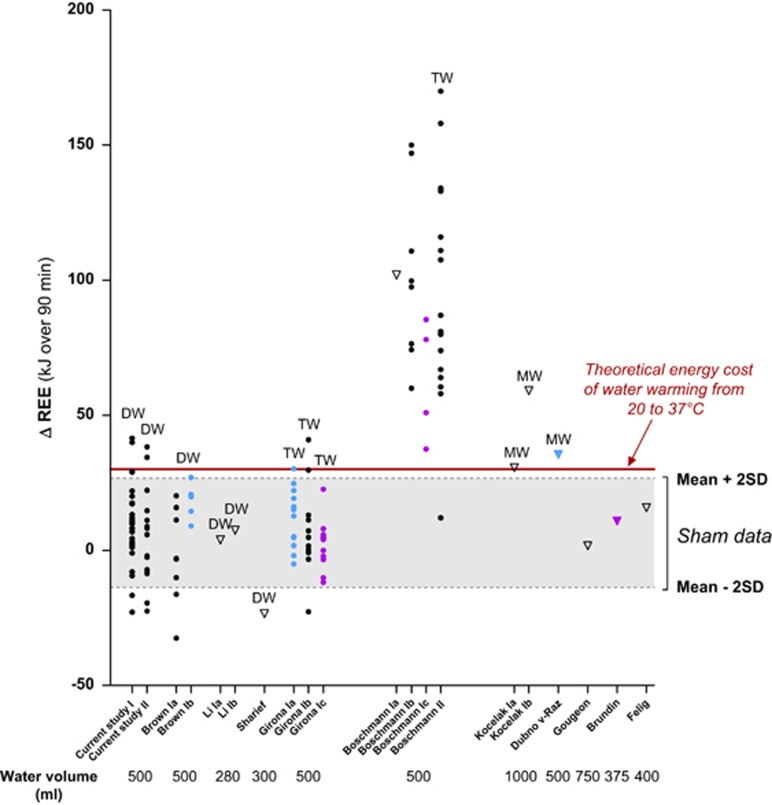
Interstudy comparisons of ΔREE (kJ over 90 min) after drinking water. Data are for 20 experiments conducted in several laboratories (see Table 1 for references), including the current study (I for data presented in main text, and II for data presented in Supplementary Information, section B). Individual values are represented as filled circles; otherwise the mean values are indicated as triangles. Water types are abbreviated as follows: TW, tap water; DW, distilled water; MW, mineral water. Unlabeled studies are those for which water type was not specified. Water temperature are indicated as follows: black for room-tempered water (20–22 °C), pale blue for cold water (3–4 °C), and purple for body-tempered water (36.5–37 °C). The grey zone represents the range of values within mean±2 s.d. of ΔREE after Sham drinking in young adults in our experiment. Note: (i) for the study of ‘Li' conducted in women, 1a and Ib values correspond to data obtained in the follicular and luteal phases of the menstrual cycle, respectively; (ii) for the studies of ‘Boschmann', Ia, Ib and Ic are for data reported in Boschman *et al.*,^6^ whereas II is for data reported in Boschman *et al.*^7^; (iii) for the study of ‘Kocelak', Ia and Ib are for data in normal weight and obese subjects, respectively; (iv) for the study of ‘Dubnov-Raz', the mean value has been calculated from data presented in a figure. The average pre-drink REE (kJ min^−1^) was calculated as the average of three data points for REE during 6 min before water drinking, and the average post-drink REE (kJ min^−1^) between 15 and 60 min. The increase in REE is then calculated as the difference between the average post-drink REE and average pre-drink REE, and then multiplied by 90 to estimate the increase in REE (kJ) over 90 min post drink.

For comparative purposes, data from published studies (Table 1) reporting the effect of water drinking on REE, are plotted in Figure 2 as the change in REE above baseline (ΔREE) over 90 min post drink. Comparison of individual experiments conducted in our laboratory reported here and previously^19, 20^ indicates that after ingestion of 500 ml of water, whether DW or tap water (TW), the absolute change in REE calculated over 90 min post drink was <20 kJ on average; as also observed in past studies from other laboratories reporting ingestion of volumes of water in the range of 280–750 ml.^10, 11, 12, 13, 14, 15, 16, 17, 18^ Examination of individual data from our current study and from our previously published work^19, 20^ indicates that even the highest values for the increases in heat production (ΔREE) over this 90 min post drink period are <50 kJ, whether in men or women, normal weight, overweight or obese, drinking room-tempered or cold water. All these values are within (or close to) ±2 s.d. of the mean value observed with Sham drinking (based on our 27 subjects) and cover 95% of data points. Consequently, the DW response cannot be attributed to effects of ingested water *per se*. By contrast, the increases in REE over 90 min post drink found in the two studies conducted in the laboratory of Boschmann *et al.*^6, 7^ using the same fixed water volume (500 ml) and same water temperature (21–22°) are uniquely spectacular. Not only does the mean increase largely exceeds even the highest individual ΔREE found in our laboratory and the mean values of other studies^10, 11, 12, 13, 14, 15, 16, 17, 18^ but most of their subjects show increases in REE between 50 and 150 kJ, with some subjects up to 150–170 kJ. These latter values are greater than that observed for the thermic effect of 75 g of glucose ingested with 500 ml of water,^32^ and several times greater than the theoretical cost of warming room-tempered (21–22 °C) water to body temperature (~30–33 kJ for 500 ml) under laboratory conditions. Overall, the findings of large increases in REE after water drinking reported by Boschmann *et al.*^6, 7^ are not supported by the current studies using similar water volume (500 ml) and temperature (21–22 °C) nor by other previously published studies.^10, 11, 12, 13, 14, 15, 16, 17, 18, 19, 20^ Possible explanations underlying the high values for water-induced thermogenesis reported by Boschmann *et al.*^6, 7^ are discussed below.

#### Methodological issues

Boschmann *et al.*^6, 7^ measured REE in their subjects by indirect calorimetry in a whole-body respiratory chamber as opposed to the use of the ventilated canopy or mouthpiece techniques.^10, 11, 12, 13, 14, 15, 16, 17, 18, 19, 20^ Ventilated hood and mouthpiece apparatus have a small dead space, thereby enabling rapid attainment of steady-state gas concentrations. By contrast, because respiratory chambers have a slower response time, and the data for REE and RQ are confounded by early activities within the chamber, they are therefore less suitable for acute measurements.^19^ In their second study of water-induced thermogenesis, Boschmann *et al.*^7^ reported that their respiratory chamber was able to follow rapid changes in energy expenditure, and that when using 20-min integration periods, the time course of the postprandial thermogenic response after ingesting a standardized test meal was virtually identical over 360 min with the respiratory chamber and a canopy system, respectively. Unfortunately, they did not provide any data to support this contention.

#### Water type and water load

Water-induced thermogenesis in Boschmann *et al.*^6, 7^ studies might also result from substances dissolved in the water. They did not state in their first report^6^ whether they used TW, bottled water or DW, but in their second report^7^ in which they found an equally high increase in REE after water, they mentioned using TW (22 °C; pH: 7.5; Na^+^: 1.5 mmol l^−1^; Ca^2+^: 3.1 mmol l^−1^), albeit without specifying whether other minerals (for example, Mg) and bicarbonates were present or not in their TW. Indeed, whether the presence of minerals in bottled water used in the studies of Kocelak *et al.*^8^ and Dubnov-Raz *et al.*,^9^ through direct effects on metabolism and/or indirect effects via altered taste and sensorial effects, could have contributed to the 10–15% increase in REE observed in their subjects cannot be disregarded. It is also possible that the stress of gastric distention and discomfort of ingesting large water loads in these two studies, namely, 1 liter in adults^8^ and up to 800 ml in 9-year-old children,^9^ could also have contributed to the increased REE. In our study, we minimized these potential sources of stress and excluded minerals and other substances in water using DW in amounts (500 ml) that none of adult subjects found to cause discomfort.

### Re-evaluation of the rationale underlying the concept of water-induced thermogenesis

Our findings here, in a relatively large group of men and women that drinking pure water failed to induce more than marginal change in REE—in any of the participants—call for a re-evaluation of the rationale underlying the concept of water-induced thermogenesis, in particular:

- Why is it that an intake of substantial water load (500 ml) that has been shown to increase circulating noradrenaline and to enhance SNS activity to skeletal muscle does not lead to significant stimulation of thermogenesis?
- Why is it that the expected increase in heat production that would be required to re-establish core temperature to about 37 °C after ingesting cool (~20 °C) or even cold water (3 °C) does not occur (when compared to Sham drinking)?

### Issue of energy cost of warming water without increased heat production

One of the arguments put forward to support the concept of water-induced thermogenesis is that the body reacts to the lower temperature triggered by ingesting room-tempered (20–22 °C) water (which is at a temperature much lower than core temperature) by increasing heat production. In fact, in Boschmann *et al.*^6^ study, drinking water at 37 °C was shown to attenuate the post-drink increase in REE by an amount that closely matched the calculated energy required to heat the water from room temperature (22 °C) to body temperature (~30 kJ for 500 ml).^6^ Although this extra amount of heat is small, as indeed the theoretical drop in body temperature (0.1 °C) that would be induced by ingesting 500 ml of water at 21–22 °C in a typical 70 kg man (total body water of 45–48 liter), it must be emphasized that the vast majority of our subjects here failed to show this small extra heat requirement as an increase in heat production. Furthermore, if the hypothesis of water-induced thermogenesis is correct, then drinking cold water should stimulate further its thermogenic effect. Although Brown *et al.*^19^ found that drinking DW that had been cooled to 3 °C slightly increased REE by 15 kJ on average over 90 min, this value is far below the 70 kJ calculated to be the energy required to heat the water from 3 to 37 °C (~500 ml × 34 °C≈17 kcal≈70 kJ). Thus, most of the energy required for warming the water to body temperature is most likely met through diminished body heat loss, probably via peripheral vasoconstriction that occurs after water drinking.^5^ This latter contention is consistent with the lower skin blood flow observed after drinking cool or cold water, but not body-tempered (37 °C) water.^20^

### Issue of sympathetic activation without increased thermogenesis

The rationale for studies investigating water-induced thermogenesis derives from earlier findings that drinking half a litre of water increases SNS activity, as measured by enhanced plasma noradrenaline levels^4^ and muscle sympathetic nerve activity.^5^ Because thermogenesis is, to a large extent, under regulation by the SNS, the notion that water drinking might also stimulate thermogenesis therefore seems plausible. It should, however, be emphasized that there is considerable heterogeneity in sympathetic activation *vis-à-vis* physiological functions. The application of noradrenaline-turnover technique to assess SNS activity in peripheral organs/tissues has shown that it is increased in the heart, liver, brown adipose tissue, kidneys and pancreas of rats in the fed relative to fasted state, but whether all these organs/tissues contribute to diet-induced thermogenesis is uncertain.^33^ Although in humans increase in sympathetic activity to skeletal muscle has been reported after water drinking,^5^ this may not necessarily translate into thermogenesis. Indeed, infusion of noradrenaline in humans results in an increase in whole-body REE, but without detectable increase in oxygen consumption in forearm skeletal muscle. In fact, the observed increase in muscle sympathetic activity after water drinking may only be influencing the muscle vasculature rather than myocyte metabolism. This contention is consistent with findings that the increase in SNS activity after water drinking is accompanied by peripheral vasoconstriction and diminished limb blood flow.^5^ Furthermore, the observed increase in circulating noradrenaline, which may reflect noradrenaline spillover from increased sympathetic neural activity to the vasculature, may not have exceeded the threshold concentration that would be required to enhance thermogenesis in myocytes or in other tissues or organs.

## Conclusions

Using the ventilated hood indirect calorimetry system, considered to be most appropriate approach to study acute changes in REE and RQ, our study comparing the ingestion of purified (distilled) water to Sham drinking suggests that water ingestion *per se* does not result in stimulation of thermogenesis or fat oxidation in healthy young men and women varying widely in adiposity.

## Figures and Tables

**Table 1:** Summary of published studies reporting acute effects of water ingestion on REE and RQ in humans

*Study (ref)*	*Year of publication*	*Water volume (ml)*	*Water type (°C)*	*No. of subjects*	*Subjects (gender and BMI)*	*Calorimetry method*	*Reported increase in REE*	*Reported change in RQ*
Girona *et al.*^20^	2014	500	Tap water (22 °C)	12	8 men, 4 women, all lean	Ventilated hood	2–3%	Transient decrease within 40 min
Kocełak *et al.*^8^	2012	1000	Low mineralized water (22 °C)	45	Women, 24 obese, 21 lean	Not specified	15% in obese; 10% in lean	No change in obese; decreased in lean
Dubnov-Raz *et al.*^9^	2011	~500 (343–798)	Mineralized local water (4 °C)	21	11 boys, 10 girls (7–12 years old), overweight or obese	Ventilated hood	25%	No
Boschmann *et al.*^7^	2007	500	Tap water (22 °C)	16	8 men, 8 women, overweight or obese	Whole room	24%	No
Brown *et al.*^19^	2006	~518	Distilled water (21 °C)	8	6 men, 2 women, all lean	Ventilated hood	No	No
Gougeon *et al.*^10^	2005	750	?	2	2 women, BMI?	Ventilated hood	No	—
Boschmann *et al.*^6^	2003	500	Water type? (22 °C)	14	7 men, 7 women, all lean	Whole room	30%	Decrease in men/increase in women
Komatsu *et al.*^11^	2003	300	Boiled distilled water (37 °C)	11	Women, BMI?	Douglas bag	No (2.7%)	No
Li *et al.*^12^	1999	280	Distilled water	19	Women, BMI?	Ventilated hood	No	—
Brundin and Wahren^13^	1993	375	Water type? (36.5 °C)	7	Men, BMI?	Ventilated hood	No (2–3%)	No
De Jonge *et al.*^14^	1991	>400	?	9	4 men, 5 women, all lean	Ventilated hood	No	Decrease
Dulloo and Miller^15^	1986	200	Tap water (22 °C)	16	8 men, 8 women (4 lean, 4 post-obese per group)	Douglas bag	No	—
LeBlanc *et al.*^16^	1984	600	?	8	Men, lean	Pneumatograph	No	—
Felig *et al.*^17^	1983	400	?	3	Women, lean	Ventilated hood	No	—
Sharief and Macdonald^18^	1982	~300	Distilled water	6	Men, lean	Ventilated hood	No (decrease)	Decrease

Abbreviations: BMI, body mass index; REE, resting energy expenditure; RQ, respiratory quotient; ?, data/information unavailable.

**Table 2:** Baseline values (mean±s.e.m.) for REE and RQ before water drink versus before sham drink; there are no significant differences in baseline values for the two test days

*Parameter*	*Sham drink*			*Water drink*		
	*All*	*Men*	*Women*	*All*	*Men*	*Women*
REE (kJ min^−1^)	4.32±0.17	5.05±0.13	3.54±0.10	4.33±0.18	5.02±0.16	3.57±0.10
RQ	0.82±0.01	0.82±0.02	0.81±0.01	0.82±0.01	0.81±0.01	0.82±0.01

Abbreviations: REE, resting energy expenditure; RQ, respiratory quotient.

## Supplementary Material

Supplementary Informations
