# Supplementary material for: Water-induced thermogenesis and fat oxidation: a reassessment
Source: Nutr Diabetes. 2015 Dec 21;5(12):e190–. doi: 10.1038/nutd.2015.41 (PMC4735055; doi:10.1038/nutd.2015.41)
Supplement: Supplementary Informations [file nutd201541x1.doc]

*Water-induced thermogenesis and fat oxidation: a reassessment*

*Nathalie Charrière, Jennifer L Miles-Chan, Jean-Pierre Montani, Abdul G Dulloo*

*Department of Medicine, Division of Physiology, University of Fribourg, Switzerland*

***Supplementary Information***

***Section A.***

*Effect of distilled water (DW) and sham drinking on resting energy expenditure (REE) and respiratory quotient (RQ): Analysis by gender*

The time course of changes in REE and RQ after Sham drink or DW (*presented in main text for 27 young adults; pooled gender*) is provided here in the **Figure SI_1** below separately for each gender: in men only (n=14; *upper panel*) and in women only (n=13; *lower panel*). The data (mean ±SE) are presented as changes relative to baseline. Repeated-measures ANOVA assessed statistical differences as follows: effect of Time (symbol †) and the Drink x Time interaction (symbol ‡); one, two and three symbols denoting p<0.05, p<0.01 and p<0.001, respectively. Significant difference between post-drink and baseline values are indicated as follows: °: p<0.05; °°: p<0.01; °°°: p<0.001).

**Figure SI_1**

**ΔREE ΔRQ**

†††

*Men*

*n=14*

††

†††

‡‡

†††

*Women*

*n=13*

***Section B.***

*Effect of distilled water (DW) on resting energy expenditure (REE) and respiratory quotient (RQ): a repeatability study*

*Aim/Design*

To increase the pool of subjects, and to assess repeatability of REE and RQ responses to water drinking, a separate experiment was conducted in 17 healthy young men, with a mean (±SEM) age of 23 ± 1 y, body weight of 77.9 ± 2.3 kg, and body mass index (BMI; kg/m^2^) of 24.2 ± 0.5 (range 21.4 to 28.1); and involved the ingestion of 500 ml of room-tempered (21-22 °C) DW. Repeatability of the metabolic responses to the water drink was assessed in 10 subjects who repeated the experiment on two different mornings, with at least 2 days interval.

*Results*

In the **Figure SI_2** below, the pre-drink (baseline) values and post-drink values for REE and RQ averaged over 130 min are presented in panel A for each subject; the bold broken line representing the overall mean change. In response to the water drink, there is a slight increase in REE of ~ 1% (not significant by paired t-test); the individual changes range between -3% to 9%, with most individuals (14 out of 17) showing <3% change in REE. By contrast, RQ is significantly reduced after water ingestion by 0.04 on average (p<0.001), with all subjects showing changes in RQ within the range of 0 to -0.07.

The results on changes in REE and RQ of the subjects who repeated the test on two separate days (Day 1 and Day 2) are shown in panel B; the diagonal dotted line represents the identity line, while the broken lines perpendicular to the x-axis and y-axis represents the mean value for Day 1 and Day 2, respectively.

Application of the Bland-Altman plot analysis (shown in panel C) to assess repeatability of the measurements indicates the following:

1. there is no significant mean bias (shown as dotted line) between measurements made on Day 1 and Day 2 for either ∆REE or ∆RQ,

1. there is no proportional bias as indicated by the lack of significant linear regression between the measurement *differences* as a function of the *average* of these two measurements, and

1. although the mean bias is not significant from zero, the within-subject variability of the difference between the two measures is larger, the standard deviation (SD) being 0.184 kJ/min for ∆REE and 0.024 for ∆RQ. Thus the 95% limits of agreement (shown as broken lines) vary between -0.356 to 0.367 kJ/min for ∆REE, and between -0.0388 to 0.0538 for ∆RQ.

The within-subject (or intra-individual) variability for ∆REE (i.e. SD of 0.184 kJ/min) is about two times the inherent analytical variability of our indirect calorimetry (Cosmed Quark) system which has a SD of 0.1 kJ/min.

**Figure SI_2**

A

C

B
